# Mycobacterial drug discovery

**DOI:** 10.1039/d0md00261e

**Published:** 2020-11-06

**Authors:** Katherine A. Abrahams, Gurdyal S. Besra

**Affiliations:** Institute of Microbiology and Infection, School of Biosciences, University of Birmingham Edgbaston Birmingham B15 2TT UK g.besra@bham.ac.uk +44 (0)121 41 45925 +44 (0)121 41 58125

## Abstract

*Mycobacterium tuberculosis* is the causative pathogen of the pulmonary disease tuberculosis. Despite the availability of effective treatment programs, there is a global pursuit of new anti-tubercular agents to respond to the developing threat of drug resistance, in addition to reducing the extensive duration of chemotherapy and any associated toxicity. The route to mycobacterial drug discovery can be considered from two directions: target-to-drug and drug-to-target. The former approach uses conventional methods including biochemical assays along with innovative computational screens, but is yet to yield any drug candidates to the clinic, with a high attrition rate owing to lack of whole cell activity. In the latter approach, compound libraries are screened for efficacy against the bacilli or model organisms, ensuring whole cell activity, but here subsequent target identification is the rate-limiting step. Advances in a variety of scientific fields have enabled the amalgamation of aspects of both approaches in the development of novel drug discovery tools, which are now primed to accelerate the discovery of novel hits and leads with known targets and whole cell activity. This review discusses these traditional and innovative techniques, which are widely used in the quest for new anti-tubercular compounds.

## Abstract 2

Innovations in mycobacterial drug discovery to accelerate the identification of new drug candidates with confirmed targets and whole cell activity.

## Graphical abstract

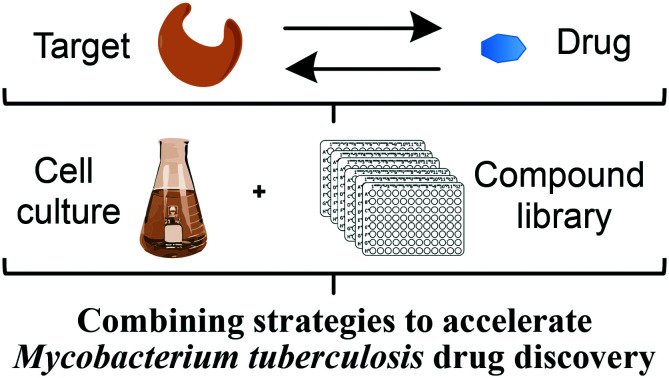

## Introduction

*Mycobacterium tuberculosis* (*Mtb*), the etiological agent of tuberculosis (TB), is the leading cause of death from a single infectious agent and ranks in the top ten causes of death worldwide. Despite innovations in diagnostics, treatment programs and healthcare provision, in 2019, this largely treatable disease infected an estimated 10 million people and was responsible for approximately 1.4 million deaths.^1^ The global burden of this disease is augmented by multi-drug resistant (MDR) and extensively-drug resistant (XDR)-TB strains, which continue to threaten the efficacy of treatment strategies and this drives the demand for the discovery of new anti-tubercular drugs to complement existing regimens.^2^ Presently, there are twenty-three anti-tubercular drug candidates in clinical trials and combination programs for the effective treatment of drug-susceptible, MDR and latent *Mtb* strains.^1^ However, continuous and concerted efforts from multiple research disciplines is paramount in the development of new clinical drug candidates, which remains at the forefront of this on-going global battle against TB.

Various considerations direct the development of new anti-tubercular agents. These compounds ideally need to be affordable, compatible in combination therapies, reduce the treatment duration (currently 6 months minimum) and have a low mutation frequency of resistance.^3^ They also need to exhibit low toxicity over long time periods with minimal side-effects or drug interactions.^1^ In addition, to these desirable characteristics, the discovery of novel hits and leads against *Mtb* alone poses an inherent challenge. *Mtb* has a slow growth rate, which negatively impacts the rate of research. Drugs targeting *Mtb* need to be active in different phases of infection; *Mtb* can exist in diverse microenvironments (including macrophages and granulomas),^4^ as well as in physiologically distinct sub-populations: replicating (aerobic conditions) and non-replicating (anaerobic conditions), as well as resistant and persistent states.^5,6^ Adding to the challenge of anti-tubercular drug discovery is the distinguishing feature of mycobacteria, an expansive and adaptable cell wall.^7^ This macromolecular structure is essential for survival and pathogenesis and serves as a permeability barrier, protecting the bacilli against hydrophilic compounds.^8^ It is therefore unsurprising that two of the drugs in mainstay TB treatment and others progressing through clinical trials, target the synthesis of key cell wall components.^9,10^ Over the past decade, developments in genomic and molecular techniques, including valuable insights gained from whole genome sequencing (WGS),^11^ has advanced our understanding of the biochemistry and pathogenicity of *Mtb*, facilitating the exploration of chemotherapeutic agents targeting novel cellular processes. Despite this, less than 0.5% of *Mtb* proteins are exploited in current treatment strategies,^12^ exemplifying the goldmine of unexploited targets. This review explores the diverse techniques in mycobacterial drug discovery with an ultimate outlook: a combined response from distinct areas of research, which should accelerate the discovery of pre-clinical drug candidates.

## Target-to-drug and drug-to-target approaches

Research into the discovery of novel anti-tubercular drugs requires a concerted response from a diverse range of fields including biochemistry, microbiology, structural biology and chemistry, informatics, physical and organic chemistry to name but a few. The drug discovery paradigm has to continuously adapt in response to technological advances in these fields whilst complementing traditional techniques. In the drug discovery pipeline, target identification, although not always possible, is a valuable step and can determine the suitability of whether a compound can progress into a pre-clinical drug candidate.^13^ Therefore, drug discovery can be approached from two main angles: target-to-drug^14^ or drug-to-target,^15^ and advances in scientific innovations dictate the equilibrium in favour of either directive, which is in constant flux. In target-based drug discovery, *in vitro* or *in vivo*, compounds are screened or developed against a pre-determined metabolic process or enzyme. This ensures target suitability, such as those lacking human orthologues, those with no known pre-existing drug resistance, favourable localisation (*e.g.* on the extracellular surface), and for many more reasons. However, the whole cell activity of the compound requires verification, and failure to establish this contributes to the high attrition rate in target-based drug discovery. In contrast, the drug-to-target approach benefits from confirmed whole cell activity of the compound, but the target requires subsequent identification. This is a challenging process that could transpire an unfavourable or uncharacterised mode of action and often results in the rediscovery of inhibitors targeting a small subset of ‘promiscuous’ targets, such as MmpL3 or DprE1.^16^ MmpL3 is responsible for transporting trehalose monomycolate across the plasma membrane for cell wall biosynthesis.^17,18^ DprE1 is also involved in cell wall biosynthesis, generating the lipid-linked arabinose precursor of the arabinan cell wall component.^19^ The high ‘hit rates’ from screening campaigns of these drug targets can be rationalised by their plasma membrane location, making them accessible to inhibitors.^20^ There is an impetus towards the complementary use of the target-to-drug and drug-to-target approaches, to benefit from the advantages and alleviating the pitfalls of each, accelerating the discovery of new drug candidates.

## Drug-to-target inhibitor discovery and target identification

**Fig. 1:**
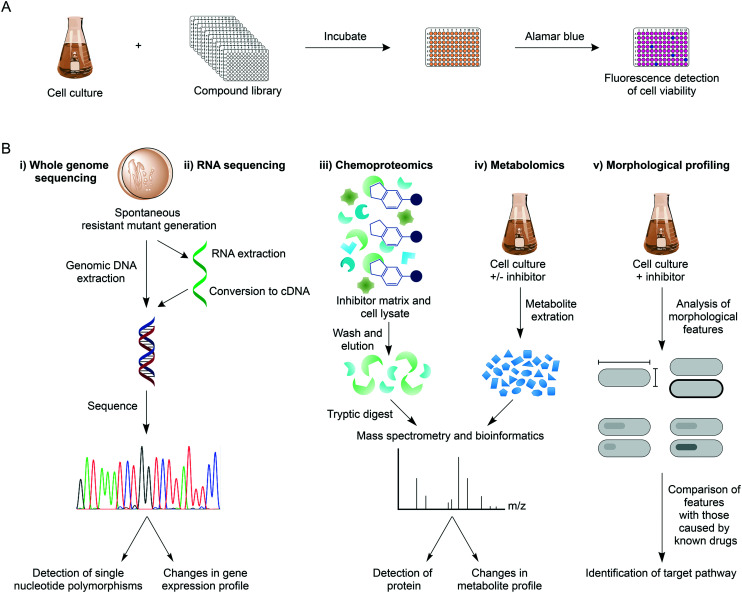
Whole cell phenotypic screening and target identification. A) Drug-to-target drug discovery generally exploits whole cell phenotypic screening, which involves the incubation of a mid-log culture of mycobacteria with a library of compounds at a specified concentration (usually 10–20 μM), in a multi-well plate format to allow for high-throughput. Cell viability can be established by the microplate alamar blue assay, where a non-fluorescent resazurin (blue) is reduced to a fluorescent resorufin (pink) by living cells. B) Following the discovery of an anti-tubercular compound, various methods can be used to establish the target. These include spontaneous resistant mutant generation against the compound followed by (i) WGS to identify resistance conferring mutations, or (ii) changes in gene expression profiles, such as the up-regulation of the target gene. Alternatively, mass spectrometry can be exploited: (iii) in a chemoproteomics approach, inhibitor-bound matrices can be used to pull down competitively binding proteins from a cell lysate; (iv) metabolomic analysis of drug-treated cell culture can be used to identify changes to the metabolome, for example the reduction of a product from an inhibited pathway. In morphological profiling (v), cells are treated with drugs that have known and unknown modes of action and the morphological features analysed. Comparisons of the two groups can reveal target pathways. This list of target identification methods is not exhaustive.

Over recent years, high-throughput screens (HTS) of extensive compound libraries have changed the fortune of TB drug discovery efforts, unearthing a plethora of diverse inhibitory molecules.^21^ With hundreds of thousands of drugs available, phenotypic whole cell HTS have been used to rapidly identify inhibitors of *Mtb*.^22,23^ These screens commonly employ the microplate alamar blue assay to assess drug susceptibility (Fig. 1A),^22–25^ but alternative methods such as monitoring cell growth through the heterologous expression of a fluorescent marker can be an alternative approach.^26,27^ The screens can be modified to mimic the different physiological states of *Mtb* during infection, such as aerobic and anaerobic conditions,^28,29^ starvation,^30^ in addition to the requirement of different carbon sources and nutrients^31,32^ and in *Mtb*-infected macrophages.^33,34^ The phenotypic-based hits discovered from these screens already circumvent permeability issues and can be further assessed for additional criteria including cytotoxicity, ultimately providing compounds with good selectivity and specificity. In hit-to-lead optimisation, target validation is a crucial proceeding step (Fig. 1B). WGS of laboratory-generated spontaneous resistant mutants is routinely used to identify mutations within the target gene, which enables the resistance phenotype.^35–38^ Target identification in this way is not always possible, with examples including: 1) if mutations occur in genes responsible for the resistance mechanism, such as the up-regulation of efflux pumps^39^ or mutations in pro-drug activators;^40,41^ 2) if there is more than one target;^42^ 3) if the target is not a protein (such as the cell wall structural targets of nisin and vancomycin); 4) if spontaneous resistant mutant generation is not possible. In these circumstances, target deconvolution becomes a challenging process, but not impossible.

RNA sequencing is a valid technology in target elucidation, where analysis of the transcriptome following drug treatment can reveal changes to the expression levels of genes (target, pathway, or compensatory). This technique is especially useful if resistant mutants harbour mutations in a gene encoding a transcriptional regulator. Transcriptional profiling of delamanid of phase III clinical trials has provided insights into its mode of action.^43^ Although delamanid has been shown to inhibit cell wall synthesis,^44^ the expression of genes coinciding with this process was negligibly impacted. Contrarily, genes responding to the effects of respiratory poisoning were up-regulated, suggesting a cause of the bactericidal activity of delamanid, although the exact mode of action remains to be determined.^43^ RNA sequencing can therefore be used to facilitate target delineation.

Chemoproteomic strategies are becoming commonplace in target identification or validation. In this technique, tagged inhibitor analogues are linked to Sepharose beads, and incubated with cell extracts in the presence and absence of inhibitor. In this competitive binding strategy, the target protein is expected to predominantly bind to the inhibitor-linked beads in the absence of inhibitor. Following bead washing and tryptic digestion, quantitative mass spectrometry analysis can be used to distinguish between proteins generically binding to beads, and those that are competitively bound. This technique is not suitable for identifying targets that are not proteins, or proteins that are unstable. Also, steric hindrance of the tagged analogue may prevent target binding. However, chemoproteomics can be used to directly establish target engagement and this technique was used to identify EchA6 as the target of the tetrahydropyrazo[1,5-*a*]pyrimidine-3-carboxamide compounds,^45^ and to validate KasA as the target of an indazole sulphonamide.^35^

Metabolomics is emerging as a key tool in mode of action studies and metabolic analysis before and after drug treatment can reveal changes in the metabolome, which can be correlated to the inhibited metabolic pathway. However, a requirement of this technique is that the target or pathway have known functions or that the inhibitor's mode of action correlates with existing drugs for comparative purposes. For example, Zampieri *et al.*, (2018) have performed a high-throughput metabolomics analysis on *Mycobacterium smegmatis* treated against a library of compounds.^46^ The metabolomic responses were compared with the metabolome profiles of *M. smegmatis* treated with drugs with known targets. Using this strategy, 70% of the compounds analysed induced responses corresponding to that of known modes of action, facilitating the delineation of the specific targets of these compounds.^46^

Morphological profiling is a new tool that has been developed to identify target pathways (DNA synthesis, RNA synthesis, protein synthesis, cell wall synthesis, respiration) of anti-tubercular agents.^47^ In this approach, a platform, morphological evaluation and understanding of stress (MorphEUS), can be used to classify targets based on imaging features such as cell shape, nucleoid shape and staining intensity. This new technique was applied to 34 anti-microbials with known modes of action, grouping the majority of them to their target pathway, highlighting the success of this application for mode of action elucidation.^47^

These target identification approaches can be used in parallel with other biochemical characterisations, including kinetic and binding assays and co-structural determinations, to validate the target. However, although favourable for the prioritisation and progression of compounds into pre-clinical drug candidates, target identification is not necessarily essential, if for example, a compound exhibits encouraging characteristics in terms of efficacy and cytotoxicity profiles. This is true for the inhibitors, delamanid^44^ and pretomanid,^48^ which are completing phase III clinical trials;^1,49^ there is evidence to suggest these pro-drugs have a multifaceted approach upon activation, targeting mycolic acid biosynthesis under aerobic conditions and release reactive NO species under anaerobic conditions, causing respiratory poisoning.^43,44,48,50,51^ Further evidence reveals that upon drug treatment, there is an accumulation of a toxic metabolite, methyl glyoxal, resulting from the build-up of sugars in the pentose phosphate pathway, suggesting an alternative mechanism of killing by these pro-drugs.^52^ Given the therapeutic potential of these drugs, the specificities of the inhibition mechanism could prove valuable in the further optimisation of these compounds.

**Fig. 2:**
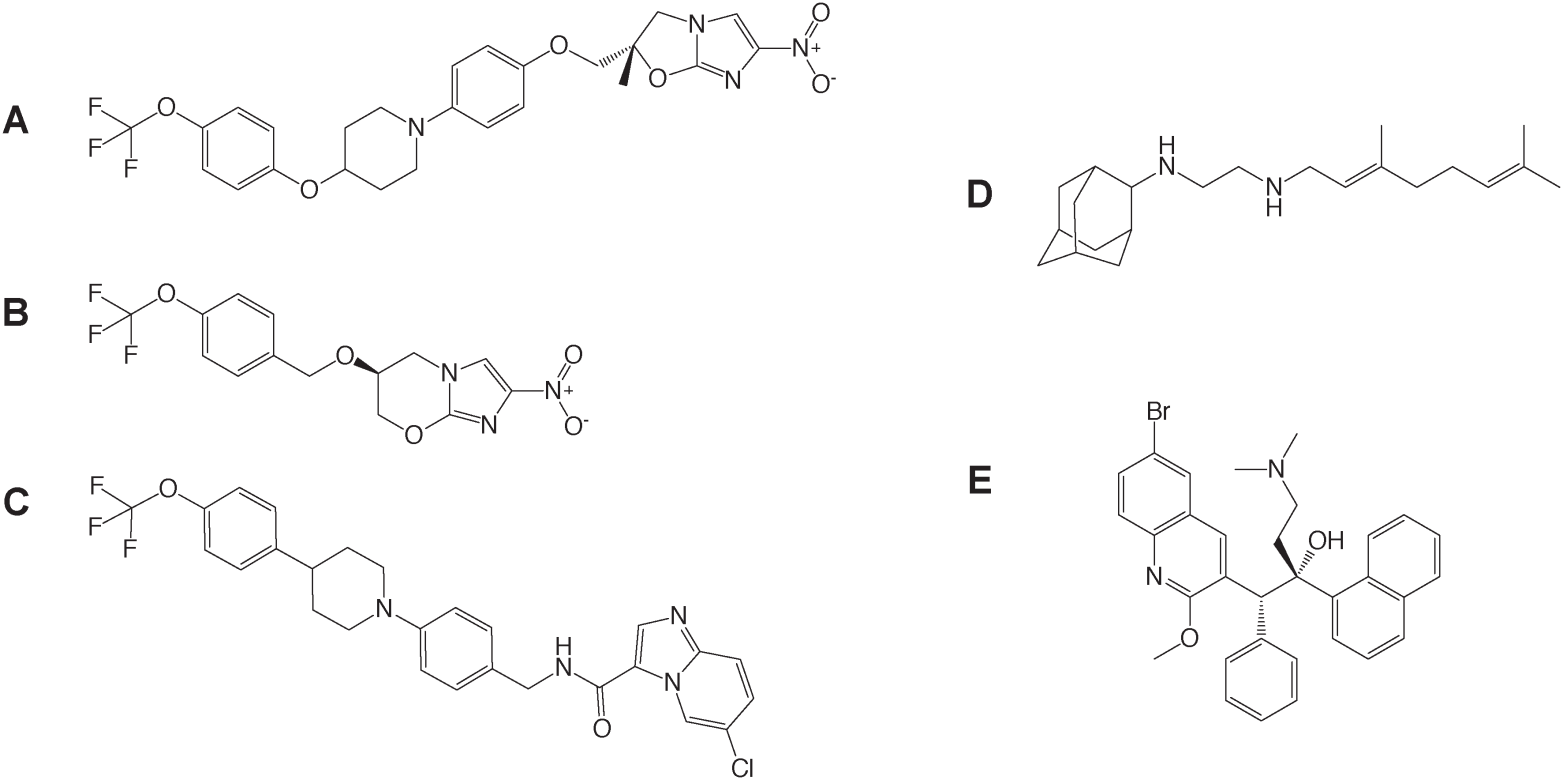
Drug candidates identified from whole cell HTS. The structures of A) delamanid, B) pretomanid, C) Q203, D) SQ109 and E) bedaquiline are shown, which are currently progressing through clinical trials.

Drug-to-target whole cell phenotypic HTS of inhibitor libraries have yielded a number of drug candidates (delamanid,^44^ pretomanid,^48^ SQ109,^53^ Q203,^34^ bedaquiline^54^) that are currently progressing through clinical trials^1^ (Fig. 2). This exemplifies the success of whole cell screening in drug discovery.

## Target-to-drug inhibitor identification

For target-based drug discovery, the landmark achievement of the sequencing of the *Mtb* genome has accelerated the discovery of new potential drug targets, with known, predicted and unidentified activities. The essentiality of *Mtb* genes, originally assessed using transposon site hybridization (TraSH), are now commonly determined by deep sequencing based approaches such as transposon sequencing (Tn-seq). In the TraSH method, a pool of transposon insertion mutants are generated, where a single *Himar1* transposon inserts into the dinucleotide TA, which are present approximately every 60 bases in the *Mtb* genome.^55^ Strains where essential genes have been disrupted by transposon insertion will be absent from the pool of transposon mutants. The surviving strains contain mutations in non-essential genes, which can be detected through probe generation and microarray hybridisation.^55,56^ In a similar approach, Tn-seq combines the generation of transposon insertion mutants with massively parallel sequencing, enabling the precise location of the insertion to be mapped onto the genome sequence.^57^ Large pools of transposon mutant strains have been analysed to identify genes that are essential for growth under different conditions.^56,58,59^ However, transposon mutagenesis has limitations for target discovery, including: 1) genes lacking the TA dinucleotide cannot be analysed; 2) site specificity renders 9% TA sites less permissible to transposon insertion leading to approximately 1% of genes to be falsely classified as essential in the TraSH methodology; 3) transposon insertions into non-essential regions in or adjacent to essential genes could falsely classify the gene as non-essential; 4) insertions into operons can impact downstream activity; 5) the essentiality of genes is determined *in vitro*, which may not be relevant during infection.^55,56,58,59^ Based on these limitations, caution must be taken when selecting a target based on its predicted essentiality. Examples include PBPA and RodA, a transpeptidase and transglycosylase, respectively, with roles in peptidoglycan synthesis^60^ and RipA, a d,l-endopeptidase involved in peptidoglycan remodelling.^61^ These enzymes exhibit differences in essentiality *in vitro versus in vivo*. Additionally, genes classified as non-essential by TraSH, such as the catalytically inactive enoyl-CoA hydratase EchA6,^45^ have been shown to be essential. Other developments in molecular biology techniques have been beneficial in the study of gene essentiality. For example, knock-out studies, including anhydrotetracycline-inducible gene expression systems,^62^ allow gene essentiality to be established *in vivo* during different stages of infection,^63^ enabling the validity of a target to be confirmed.

With the ever increasing availability of biomedical data, for example from gene expression profiles and proteomics, data mining has further led to an increase in the identification of valid targets. The selection of a gene for target-based drug discovery efforts ultimately requires the druggability of the target to be confirmed (essentiality, redundancy, vulnerability during infection, homology to human proteins, mutation rates, activity during dormancy, to address a few criteria).^14^ This is to avoid wasting copious time and resources focusing on the development of inhibitors against an unsuitable mode of action. In TB drug discovery, validated druggable targets equate to a very small number, exemplifying the need for new targets to circumvent problems associated with the treatment of MDR and XDR-TB.

To advance the discovery of novel putative targets, an *Mtb*-specific protein druggability database (TuberQ) has been developed.^64^ Using information from unique *Mtb* structures and homology-based models, the structural druggability of a given protein is determined through the identification and analysis of putative inhibitor binding pockets. This data is combined with the physiological relevance of proteins (gene essentiality and expression under different stress conditions), enabling the rapid inspection of target suitability based on the structural and biological druggability of a chosen protein.^64^ This tool, therefore, is highly valuable in target-based drug discovery.

**Fig. 3:**
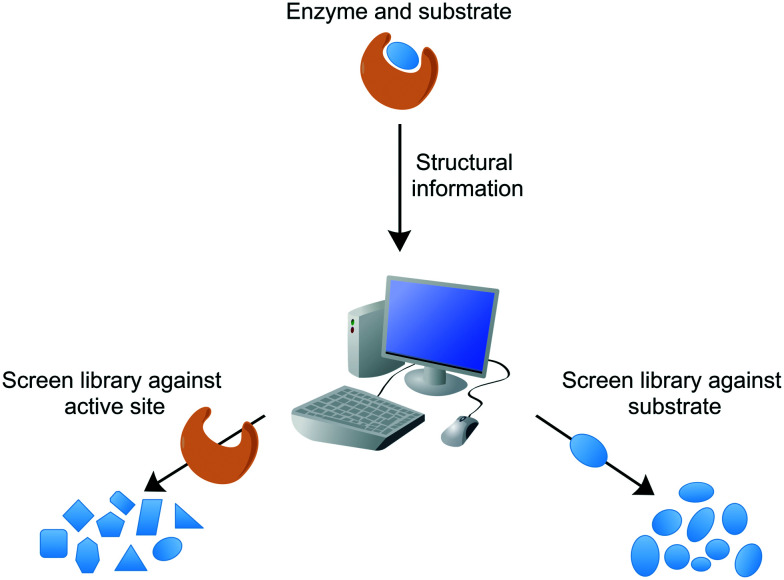
*In silico* drug discovery. Structure-based or ligand-based drug discovery involves the computational screening of a compound database against the protein target structure (*e.g.* active site) or the ligand (*e.g.* substrate, transition state or product analogues).

Conventional target-based drug discovery typically employs the development of *in vitro* biochemical assays that demonstrate biological activity. The assays rely on the availability of purified recombinant protein and suitability of substrates (or appropriate analogues) or products for kinetic characterisation in the presence of potential inhibitors. Technological developments over the past two decades have enabled the adaptation of single biochemical assays into a high-throughput format, for the rapid analysis of compound libraries. Modern target-based drug discovery techniques exploit the recent advances in 3D-structural determinations, courtesy of X-ray crystallography, nuclear magnetic resonance (NMR), and cryogenic electron microscopy (cryo-EM), in addition to computational or *in silico* analytical methods, which have facilitated the rational design of inhibitors or virtual screening of compound libraries against the target.^65^ Both of these methods can be divided into either structure-based or ligand-based, searching for inhibitors against the target active site or similarities with the known ligand, respectively (Fig. 3). For example, inhibitors of *Mtb* dihydrofolate reductase have been identified by two distinct ligand-based approaches and complemented by a structure-based approach (DHFR).^66^ Other structure-based approaches include the discovery of new inhibitors of InhA and DprE1, enzymes involved in cell wall biosynthesis.^67,68^*In silico* studies are valuable not only in the search of new inhibitors, but also in the structural optimisation of existing drug candidates to improve pharmacokinetic and pharmacodynamic properties. These techniques can provide a vast quantity of potential inhibitors. However, the attrition rate for target-to-drug based inhibitor discovery is high, owing to problems associated with drug entry into the cell, toxicity, poor pharmacokinetic properties, and complex synthetic pathways. Screening compounds with known inhibitory activity against *Mtb* can alleviate some of these difficulties.

Fragment-based drug discovery is another useful tool in the identification of target-specific small molecule inhibitors. In this approach, a library of diverse fragments, often developed by computer simulations for a selected target, is screened against a target protein. Various techniques can be used to determine fragment interaction with the target protein, including differential scanning fluorimetry,^69^ saturation transfer difference (STD)-NMR,^70^ and surface plasmon resonance,^71^ in addition to the more traditional biochemical assays. For example, a 1360 fragment library has been screened against NADH-bound InhA. Primarily, STD-NMR was used to identify fragment binding, and hits were then further confirmed through biochemical assay and SPR.^70^ Fragments from such screens can be further developed into lead compounds.

## Combining drug-to-target and target-to-drug approaches

**Fig. 4:**
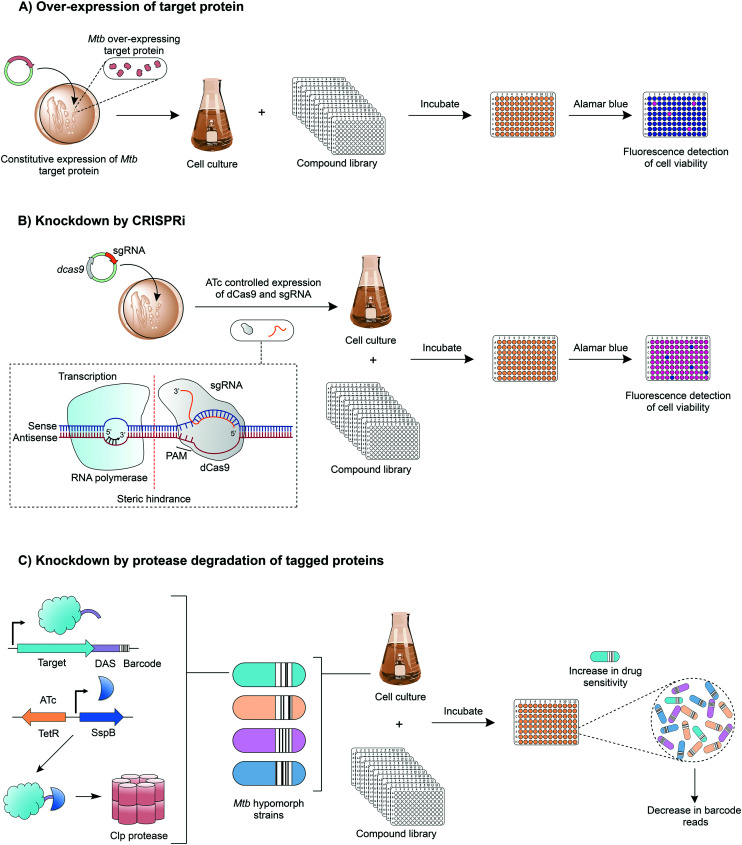
Drug discovery techniques combining drug-to-target and target-to-drug approaches. A) Target-based whole-cell phenotypic screening. In this technique, a compound library is screened against cells over-expressing a target protein (from a mycobacterial vector). Assessing cell viability at a fixed drug concentration enables the comparison of drug sensitivities between strains with and without the over-expression of the target protein and can reveal inhibitor-target engagement. B) Target protein knockdown by CRISPRi. In this approach, target protein expression is reduced by interfering with the transcription of mRNA. Anhydrotetracycline (ATc) regulates the transcription of a small guide RNA (sgRNA) and a catalytically dead endonuclease, dCas9. The sgRNA contains a short complementary sequence to the target gene and a region for dCas9 recognition. A proto-spacer adjacent motif (PAM) is essential for dCas9 binding. The sgRNA–dCas9 complex anneals to the target sequence, adjacent to PAM, blocking transcription by RNA polymerase, and depleting the expression of the target protein. C) Target protein knockdown by protease degradation allows the reduction of a specific protein through targeted degradation. In this specific approach, a degradation tag (DAS) and a barcode are integrated onto the 3′ end of the target gene. ATc drives the expression of SspB, which recognises the DAS tag and shuttles the protein to a Clp protease. In a drug screen, a strain will exhibit increased inhibitor sensitivity if the degraded protein is the drug target. The specific strain can be detected from a mixed culture of hypomorphs through quantitative sequencing of the barcode.

Innovative strategies are being developed to combine the advantages of drug-to-target and target-to-drug approaches and to circumvent any pitfalls. Target-based whole cell phenotypic HTS enable the rapid, simultaneous discovery of new whole cell-active inhibitors with known modes of action. An example is the use of mycobacteria over-expressing a target gene, which can be used to screen compound libraries (Fig. 4A); increased resistance to a compound signifies target engagement, where the additional target protein or enzyme can compensate for the inhibition of that of the native activity, or alternatively the additional target can saturate with inhibitor, enabling the native protein to function. This technique is exploited in drug discovery and target validation strategies. For example, the over-expression of DprE1^72^ and GuaB2 (inosine monophosphate dehydrogenase)^73^ have led to the discovery of novel inhibitors specifically targeting these enzymes; over-expression studies of KasA (β-ketoacyl synthase),^35^ QcrB^36^ (subunit b of the cytochrome bc_1_ complex) and TrpAB (tryptophan synthase) have confirmed target engagement as predicted by WGS of resistant isolates.^37^

A relatively new tool with applications in target-based whole cell phenotypic screening is clustered regularly interspaced short palindromic repeat interference (CRISPRi),^74^ which enables the tuneable knockdown of proteins by interfering with gene transcription (Fig. 4B) and is applicable for both essential and non-essential genes. In this system, a small guide RNA, specific to the target gene promoter region or open reading frame, simultaneously binds to the genomic DNA and to a catalytically dead form of RNA-guided DNA endonuclease (dCas9), which results in a complex that interferes with the transcription of the target gene through steric hindrance. Through the temporary suppression of gene transcription, and ultimately the expression levels of the target protein, it is possible to analyse the impact of the gene, both *in vitro* and *in vivo*. This allows the suitability of the expressed protein as a drug target to be established, and can also be used to determine whether drug effects are mimicked, enabling predicted drug targets to be validated. Reduction of gene expression levels in the presence of inhibitors can reveal target-inhibitor relationships in a similar manner to the over-expression studies, but with the opposite result: limiting target gene expression increases sensitivity to the inhibitor.

Another exciting chemical-genetics tool has been developed for concomitant discovery of inhibitors and their target. This approach regulates gene expression through the specific degradation of tagged-proteins by caseinolytic (Clp) proteases. Johnson *et al.* (2019) have recently optimised this approach for the high-throughput screening of compound libraries against large pools of tagged proteins (Fig. 4C).^75^ Briefly, a DAS-tag and a unique barcode are introduced on to the 3′ end of a gene, creating a hypomorph, pools of which can be generated. The inducible and tuneable expression of a vector encoded stringent starvation protein B (SspB) shuttles the tagged target protein to a Clp protease where it is degraded. Upon exposure to a library of compounds, inhibitors targeting a specific hypomorph strain within a pool can be identified through barcode counts, where a decrease in signal signifies increased sensitivity to drug treatment.

Computational innovations combining structural bioinformatics, molecular modelling and systems biology can be used to identify drug–target interaction networks. For example, Kinnings *et al.* (2010) have integrated these computational strategies to construct a drug–target network, identifying drug–target interactions between the known structural proteome of *Mtb* with structurally characterised approved drugs.^12^ The rational behind this work was to identify novel pharmaceutical targets and concomitantly repurpose known drugs to treat TB, alleviating the time and investment in developing novel compounds.^12^ This network can be exploited in the analysis of any new approved drugs, with the potential to rapidly progress drugs through the drug discovery pipeline. The *in vivo* efficacy of these drugs, however, remains to be assessed, but can be circumvented by testing the approved library prior to screening. Alternatively, ‘hits’ can be re-engineered to improve potency. For example, the approved laxative and osteoarthritis compound rhein exhibits anti-microbial activity, but not against *Mtb*. To circumvent potential issues with target accessibility, the rhein scaffold has been successfully re-engineered to increase lipophilicity (enabling permeation of the hydrophobic cell wall), dramatically improving potency.^40^

With the extensive quantities of publicly available data generated from drug screening programs, *in vivo* and *in vitro*, machine learning models are rapidly being developed for data curation.^76^ These models can be used to prioritise compounds for drug discovery efforts, predicting molecules that combine activity with suitable physiochemical and pharmacokinetic properties. Machine learning can also be applied for mode of action determination, based on known drug-target interactions,^77^ as well as for the predication of resistance conferring mutations.^78^ The machine learning models are developed with the aim of increasing the efficiency of drug discovery.

## Inhibitor and target validation

Irrespective of whether an inhibitory compound has been identified through a drug-to-target or target-to-drug approach, the alternative strategies described can additionally be used to complement findings and validate a compound's mode of action. For example, it is commonplace to identify the target of a compound through WGS of resistant isolates, with an accompanying mode of action confirmation through an unexhaustive list of techniques, including biochemical and binding assays, over-expression studies, chemoproteomics and 3D structural information, to mention a few. This evidence in turn, can be used to direct medicinal chemistry efforts to optimise the compound structure (potency, toxicity, physiochemical and pharmacokinetic properties) based on structure–activity and structure–property relationships. The compounds can then be re-analysed to confirm target engagement. For example, an indazole sulphonamide from a GlaxoSmithKline directed phenotypic screening campaign^22,23^ had attractive properties for early stage drug discovery based on its potency and *in vitro* and *in vivo* profiling.^35^ Consequently, this compound was subject to target identification efforts.^35^ Initially, WGS of resistant isolates in both *Mtb* and *Mycobacterium bovis* BCG revealed SNPs in *kasA*, which encodes the essential β-ketoacyl synthase involved in the biosynthesis of mycolic acids, which are major components of the *Mtb* cell wall. Target validation techniques subsequently confirmed target engagement of the compound with KasA. Radioactive biochemical assays were performed with whole cells and confirmed the dose-dependent depletion of α- and keto-mycolic acid methyl esters consistent with the inhibition of mycolic acid biosynthesis. In a second radioactive biochemical assay using purified recombinant protein, the specific inhibition of recombinant KasA activity was demonstrated. *M. bovis* BCG strains over-expressing enzymes involved in mycolic acid biosynthesis confirmed that only KasA over-expression resulted in an increase in resistance to the indazole sulphonamide. Direct binding of the indazole sulphonamide to KasA was established through a chemoproteomics strategy. In this approach, a tagged analogue of the compound was linked to Sepharose beads and incubated with *M. bovis* BCG extracts in the presence and absence of the unbound indazole sulphonamide. Bound proteins were analysed by mass spectrometry, revealing the specific binding of KasA to the compound-derived beads. Finally, the molecular details of the structure–activity relationship between KasA and the indazole sulphonamide were determined through the analysis of a co-crystal structure. Lead optimisation efforts of this compound were pursued, but were rendered unsuccessful owing to the detection of a mutagenic aniline metabolite that could not be eliminated due to the limited structure–activity relationship (SAR) space.^79^ This exemplifies the copious efforts and resources expended in drug discovery and provides one of an infinite number of reasons as to why promising drug candidates fail to progress through the drug discovery pipeline.

A success story can be represented by the imidazopyridines. Through WGS, this compound class was shown to inhibit QcrB,^36^ a constituent of the respiratory chain cytochrome bc_1_ complex. Lead optimisation of imidazopyridine analogues, led to the discovery of Q203,^34^ which was also confirmed to target QcrB and is now progressing through phase II clinical trials.^1^ Successful drug discovery therefore relies on a combination of diverse methods, from the first instance of discovering an inhibitory compound, to validation and informing future developments. This ensures that only those compounds that meet the stringent requirements of a drug progress through to the clinic.

## Conclusion

Over the past two decades, efforts in mycobacterial drug discovery have increased exponentially, coinciding with the availability of the *Mtb* genome sequence and the urgent requirement of new drugs to combat MDR and XDR-TB. The availability of extensive small molecule compound libraries, along with the validation of a plethora of druggable targets, have accelerated the discovery of potential inhibitors from both drug-to-target and target-to-drug based screens. Innovations in combining these two opposing directives are anticipated to drive the rapid discovery and validation of new targets with the concurrent delivery of vast diverse chemical scaffolds for progression into drug candidates. Ultimately, with the current global impetus towards *Mtb* drug discovery, the future of TB drug discovery looks promising.

## Conflicts of interest

There are no conflicts to declare.
